# Epidemiology and characteristics of cases with monkeypox virus clade I in the WHO European Region, 2024 to 2025

**DOI:** 10.2807/1560-7917.ES.2026.31.24.2500955

**Published:** 2026-06-18

**Authors:** Ioannis Karagiannis, Jeffrey Pires, Pana Akhmetniyaz, Olivier le Polain de Waroux, Bart Hoorelbeke, Bertrand Gagnière, Klaus Jansen, Chrysa Tsiara, Derval Igoe, Maria Rosaria Campitiello, Catharina van Ewijk, Vítor Cabral Veríssimo, Laura Santos Larrégola, Erik Sturegård, Céline Gardiol, Taliha Karakök, Gareth J Hughes, Marc-Alain Widdowson

**Affiliations:** 1 Health Security Division, WHO Regional Office for Europe, Copenhagen, Denmark; 2 WHO Health Emergencies Programme, WHO headquarters, Geneva, Switzerland; 3 DG Preparedness and Response, Federal Public Service – Health, Food Chain Safety and Environment, Brussels, Belgium; 4 Santé Publique France, Rennes, France; 5 Department for Infectious Disease Epidemiology, Robert Koch Institute, Berlin, Germany; 6 National Public Health Organisation, Athens, Greece; 7 Health Service Executive (HSE) – Public Health: National Health Protection Office, Dublin, Ireland; 8 Head of the Department of Prevention, Research and Health Emergency, Ministry of Health, Rome, Italy; 9 National Institute for Public Health and the Environment (RIVM), Bilthoven, the Netherlands; 10 Directorate of Information and Analysis, Directorate-General of Health, Ministry of Health, Lisbon, Portugal; 11 Public Health Unit, Western Lisbon Local Health, Cascais, Portugal; 12 Coordinating Centre for Health Alerts and Emergencies, Ministry of Health, Madrid, Spain; 13 Department of Communicable Disease Control and Preparedness, Public Health Agency of Sweden, Solna, Sweden; 14 Department of Translational Medicine, Faculty of Medicine, Lund University, Malmö, Sweden; 15 Section for Infection Control and Response Measures, Communicable Diseases Division, Federal Office of Public Health, Bern, Switzerland; 16 Department of Communicable Diseases, General Directorate of Public Health, Ministry of Health, Ankara, Türkiye; 17 Field Services, UK Health Security Agency, Leeds, United Kingdom

**Keywords:** mpox, monkeypox virus, clade I, imported infections, household transmission, men who have sex with men, Europe, public health surveillance

## Abstract

**BACKGROUND:**

A public health emergency of international concern (PHEIC) was declared in August 2024 following a sharp increase in mpox cases linked to the emergence of monkeypox virus (MPXV) clade Ib.

**AIM:**

We aimed to characterise imported and locally acquired mpox clade I cases reported in the WHO European Region (WHO/Europe) since August 2024 and assess transmission patterns and response measures.

**METHODS:**

We collected data on MPXV clade I infections reported by countries in WHO/Europe through the International Health Regulations. We contacted reporting countries to collect age, sex, travel history, most likely route of transmission, clinical course, contacts and type of exposure, and implemented control and prevention measures.

**RESULTS:**

Between 14 August 2024 and 23 November 2025, 82 cases of MPXV clade I infection were reported; 45 were imported and 37 were infected in reporting countries. Seventy-nine were typed as clade Ib and two as clade Ia. Most imported cases reported heterosexual (n = 26) or close physical contact (n = 5) as possible exposure. Secondary transmission occurred in six households. One healthcare worker was infected. Since October 2025, further 17 autochthonous male cases were most likely infected through sex with other men.

**CONCLUSION:**

Imported cases of MPXV clade I infection were associated with limited household transmission. The increase in autochthonous infections among men with recent sexual contact with other men suggests undetected spread in Europe, that may become sustained. Continued surveillance, case and contact investigation are needed to understand MPXV clade I epidemiology and drivers of MPXV clade I transmission in Europe.

**Key public health message**

**What did you want to address in this study and why?**

We wanted to describe cases infected with a monkeypox virus (MPXV) variant (clade I) reported in the WHO European Region since 2024. The aim was to describe people who acquired the infection in Europe and those who acquired it outside of it, and how they got infected. This was needed to inform risk assessment on whether clade I could become established in Europe.

**What have we learnt from this study?**

We identified 82 clade I infections, most of them imported from African countries with ongoing outbreaks, but 37 acquired in Europe, including seven children and adolescents and one healthcare worker. In six instances, household members became infected. Since October 2025, however, an increasing number of infections have been reported among men with recent sexual contact with other men.

**What are the implications of your findings for public health?**

Rapid case finding, testing and contact tracing remain important to limit household and healthcare-associated spread and to contain clusters linked to sexual contact between men. Inclusive, non-stigmatising risk communication to travellers, migrant communities, men who have sex with men and healthcare workers will be key to reducing the chance of wider clade I circulation in Europe.

## Introduction

Two major genetic clades of monkeypox virus (MPXV) have been described: clade I, historically associated with Middle Africa, and clade II, historically associated with Western Africa [1,2]. Before the recent multi-country outbreaks, cases outside Africa were rare and associated with little or no onward transmission [2,3].

In 2020, the largest recorded outbreak of mpox due to MPXV clade I occurred in the Democratic Republic of the Congo [4]. In 2022, a global mpox epidemic caused by MPXV clade II prompted the World Health Organization (WHO) Director-General to declare a Public Health Emergency of International Concern (PHEIC) [5]. The monthly number of reported global cases peaked at ca 30,000 cases in August 2022 [6], followed by a decline in cases globally and in Europe [7], and in May 2023 the PHEIC was lifted [8]. Since September 2023, the Democratic Republic of the Congo had seen a sharp increase in the reported number of mpox cases, linked to both the emergence of a distinct new strain, MPXV clade Ib, and a rise in cases caused by infection with previously circulating MPXV clade Ia [9,10]. Earlier evidence, including a 2022 meta-analysis, suggested that MPXV clade I may be associated with higher case fatality than clade II [9,11]. The newly identified clade Ib rapidly spread to neighbouring African countries and elsewhere. Initial reports from the Democratic Republic of the Congo indicated sustained person-to-person transmission, including through heterosexual sex. For this reason, WHO declared mpox a PHEIC for a second time on 14 August 2024 [12]. The PHEIC was lifted in September 2025 [13]. The first case of MPXV clade Ib infection outside of Africa was reported by Sweden on the next day [14,15]. Between January 2024 and 7 November 2025, 17 countries in the WHO African Region and 26 countries in all other regions reported MPXV clade Ib cases [6], with the WHO European Region reporting most MPXV clade I cases. Evidence from imported cases [16,17] and outbreaks in Africa [10,18] indicates that MPXV clade I transmission may occur both within and outside sexual networks, including through skin-to-skin contact [19].

Rapid investigation of MPXV clade I cases and timely public health response are essential to prevent further spread, provide key data on the epidemiology of MPXV clade I and inform risk assessments for Europe and comparable settings worldwide. In this paper, we describe MPXV clade I cases reported in the WHO European Region since August 2024 to gain a better understanding of the characteristics and epidemiology of MPXV clade I cases in Europe.

## Methods

### Data collection

The WHO Office for the European Region collected information on MPXV clade I cases reported in line with WHO standing recommendations for mpox [20] through International Health Regulations (IHR) channels [21] between 14 August 2024 and 23 November 2025. Reporting countries were contacted either by email or teleconferences together with relevant colleagues from the European Centre for Disease Prevention and Control (ECDC), using a standard set of questions to collect information on age, sex, travel history, likely route of transmission, clinical course and hospitalisation, contacts, and implemented control and prevention measures.

### Case definitions

Confirmed cases were defined as persons with or without symptoms, with laboratory confirmation of MPXV clade I by molecular detection in any clinical specimen, including lesion swabs and upper respiratory tract specimens. Probable cases were defined as persons with symptoms compatible with mpox who were epidemiologically linked to a confirmed case, but without laboratory confirmation of MPXV clade I.

Cases were classified as imported if their personal history indicated that they had contracted infection outside the reporting countries in the 21 days preceding symptom onset during national case investigation activities or, otherwise, as autochthonous. Clusters of cases were defined as individual cases who were epidemiologically linked.

### Laboratory confirmation

Laboratory confirmation and clade classification were performed by national or subnational laboratories in the reporting country as part of routine public health investigation. Subclade assignment (Ia/Ib) was based on analyses by national laboratories and, where available, sequencing or clade-specific molecular characterisation. Laboratory approaches were not standardised across countries. Published descriptions of microbiological methods for selected European cases are available elsewhere [14,17,22-24].

### Statistical analysis

We described cases by age, sex, travel history, possible exposures, most likely route of transmission, hospitalisation and clinical course, care-seeking behaviour, diagnosis delays, HIV status or other clinical comorbidities that weaken the immune system, prior vaccination for mpox, number of contacts, and information on implemented control and preventive measures. Continuous variables across case groups were compared using the Kruskal-Wallis test using Stata version 19.5 (StataCorp, College Station, the United States (US)). Percentages have been rounded to the nearest decimal.

## Results

As of 23 November 2025, 13 of the 53 countries in the Region reported one probable and 81 confirmed cases of MPXV clade I infection to the WHO Regional Office for Europe; one case reported by Türkiye and one reported by Ireland were typed as clade Ia, and the remaining 79 were typed as MPXV clade Ib; the MPXV clade Ia case reported by Ireland has been described elsewhere [22]. The other, a probable case reported by Ireland, was imported and had close contact with a subsequently confirmed case of MPXV clade Ib infection. One case with travel history to Asia initially notified as MPXV clade Ib was later found to be a recombinant virus containing elements of both clade Ib and IIb [23]. 

### Cases overall

Of the 82 cases, 45 (54.9%) were imported and 37 (45.1%) were autochthonous. Likely countries or geographical regions of infection included Uganda (n = 11), the Democratic Republic of the Congo (n = 6), Rwanda (n = 5), Tanzania (n = 5), Kenya (n = 3), the United Arab Emirates (n = 3), Thailand (n = 2), the Netherlands (n = 2), Malaysia (n = 1), Pakistan (n = 1), Viet Nam (n = 1), Angola (n = 1), Eastern Africa (n = 1) and Middle Africa (n = 1). One case had travelled to Rwanda, Uganda and Tanzania within 21 days before symptom onset, and one case had travelled to both Egypt and the United Arab Emirates, but case history suggests the transmission most likely occurred in the latter. Of the 37 cases infected in Europe, 17 were secondary cases of seven imported cases or an already reported autochthonous case, and a further 20 cases were not related to any previously reported case (Figure).

**Figure:**
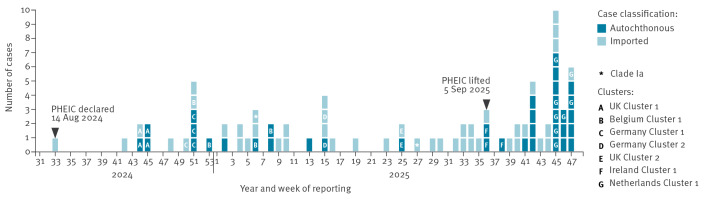
Reported autochthonous (n = 37) and imported (n = 45) cases and clusters with monkeypox virus clade I, WHO European Region, 14 August 2024–23 November 2025

PHEIC: Public Health Emergency of International Concern; UK: United Kingdom; WHO: World Health Organization.

Of the 45 imported cases, 43 were males and two were females. Age was known for 38 cases, while only the 10-year age group was known for another six; age information was missing for one case. The median age was 36.5 years (range: 18–64 years). The most likely route of infection was assessed as sexual contact for 26 of 45 imported cases, other intimate physical contact (e.g. massage, sauna services) for five and unknown for the remaining 14 cases (Table 1). Among the 25 imported male cases reported to have acquired infection sexually, the sex of the most likely infecting partner was known for 17; in 12 cases the partner was female and in five the partner was male. One additional imported case with sexual exposure was female.

**Table 1:** Reported probable mode of transmission of cases with monkeypox virus clade I, WHO European Region, 14 August 2024–23 November 2025 (n = 82)

Probable mode of transmission	Imported cases (n = 45)		Autochthonous cases (n = 37)				All cases (n = 82)	
	n	%	Household cases (n = 13)		Other cases (n = 24)		n	%
			n	%	n	%		
Sexual contact	26	57.8	4	30.8	19	79.2	49	59.8
Other contact in household	0	0	9	69.2	0	0.0	9	11.0
Close physical contact (e.g. sauna, spa, massage)	5	11.1	0	0	0	0.0	5	6.1
Healthcare setting	0	0	0	0	1	4.2	1	1.2
Unknown	14	31.1	0	0	4^a^	16.7^a^	18	22.0

^a^ One of these cases reported contact with family members who had travelled to an outbreak-affected country in Middle Africa.

Information on hospitalisation was available for 79 cases, of whom 18 (22.8%) were hospitalised; four, among whom one child, were admitted for clinical reasons and 13 for isolation or other non-mpox-related reasons. The reason for hospitalisation was not available for one hospitalised case. Prior vaccination status was available for 51 cases, among whom four had been vaccinated before symptom onset. Information on the exact vaccine product was not available. Status for HIV was known for 44 cases, among whom six were living with HIV. Information on immunocompromising conditions other than HIV was available for 36 cases, none of whom were reported to have weakened immune systems. All cases have recovered.

### Autochthonous cases

Of the 37 autochthonous cases, 25 were males and 10 were females; sex information was unavailable for two cases. Thirteen were household contacts of imported cases. Of these 13 secondary cases in households, seven were below the age of 15 years (three were aged 1–4 years, two were aged 5–9 years, and the remaining two were aged 10–14 years). Four of the 13 household cases were partners of index cases (one of whom was a probable case) and were reported to have contracted mpox sexually from their partners; the remaining nine likely acquired infection through a household contact. One of the cases infected in Europe was a healthcare worker who had cared for a hospitalised case before mpox was included in the differential diagnosis. Standard infection prevention and control (IPC) precautions appropriate to the initial working diagnosis were used, including gown/apron, gloves and a surgical mask; no breach of personal protective equipment (PPE) or needlestick injury was reported. Contact and airborne-based precautions were introduced after mpox was suspected (Table 1) [24].

Of the 24 autochthonous cases with no clear exposure to a reported case, one had no travel history but reported contact with her male partner and another family member who had both returned from a Middle African country in the previous 21 days; neither of them had reported symptoms compatible with MPXV infection. The exact source of infection for the other cases remains unknown (Table 1). Among 44 male cases who contracted mpox sexually, information on the sex of the most likely infecting partner was available for 40 of them; among these, 21 reported having most likely acquired the infection from other males, most likely in the Netherlands (n = 11), Spain (n = 8), Malaysia (n = 1) and the United Arab Emirates (n = 1). No males who acquired MPXV clade Ib through sexual contact with other males were reported to WHO before October 2025. Six of the 11 cases reported by the Netherlands and one reported by Belgium reported having attended the same sex venue in Amsterdam. Of these six cases reported by the Netherlands, four attended the venue on the same date, and the remaining two on two different dates. According to the national investigation, all six had attended within one incubation period before symptom onset, suggesting that they most likely acquired infection there.

### Imported cases

Of the 45 imported cases, information on whether the individual was returning to a household with other residents was available for 34. Of these, 25 returned to households with other residents. Transmission was identified in six of these 25 households. Within these six households, 13 additional cases occurred among 33 household contacts (overall attack rate: 39.4%; range: 0–100%). Two household cases were determined to be possible second-generation transmission events. In one household, the case had symptom onset in January 2025 following two cases with symptom onset in the same household in December 2024, although transmission through fomites or an unidentified fourth household case could not be excluded. On another occasion, a probable imported case infected one household member sexually (attack rate: 12.5%; 1/8 susceptible household contacts), who then likely infected one of the remaining seven susceptible household members through close physical contact (secondary attack rate: 14.3%, 1/7). Of 37 imported cases with information available, 15 travelled to Europe by air while having a rash and, in at least one case, the rash was on their arms and uncovered.

### Follow-up of contacts and subclade assignment

Countries reported that contacts of mpox cases were identified and risk classified in line with WHO [25] or national guidance. However, follow-up of travel-related contacts was not implemented consistently across countries. For one imported case, national health authorities asked the airline to contact the co-passengers who had been exposed to the case on a 1-hour flight. Information on the number of contacts in travel and school settings was sparse, and no secondary case was reported among travel or school contacts.

Where information was available (n = 68), subclade assignment was reported at a median of 9.5 days after symptom onset (range: −1 to 74 days); two patients were diagnosed before symptom onset due to active follow-up following household exposure. In these cases, MPXV was detected by PCR from upper respiratory tract specimens and, where lesions subsequently appeared, lesion specimens. However, specimen source and testing approaches were not systematically available for all contacts across countries.

Contacts of already reported cases sought or received care quicker and were diagnosed sooner than imported cases or those autochthonous cases with no reported exposure to a known case (Table 2). Nonetheless, contacts of known cases still took a median of 4 days after symptoms onset to seek care.

**Table 2:** Time to seeking healthcare and to diagnosis of reported cases with monkeypox virus clade I, WHO European Region, 14 August 2024–23 November 2025 (n = 72)

Case characteristics	n^a^	Median delay from symptom onset to seeking care (n = 52)		Median delay from seeking care to subclade assignment (n = 49)		Median delay from symptom onset to subclade assignment (n = 68)	
		Days	Range	Days	Range	Days	Range
Imported cases	29	5.0	1–20	6.0	0–70	11.0	2–74
Autochthonous cases with contact to already reported cases	7	4.0	0–9	5.0	2–7	6.0	−1–14
Autochthonous cases without contact to already reported cases	12	3.0	2–15	4.5	1–12	9.0	4–21
All cases	48	5.0	0–20	6.0	0–70	9.5	−1–74
Kruskal-Wallis test p value	NA	0.3350		0.5132		0.0064	

NA: not applicable.

^a^ Only cases with information about date of symptom onset, date of seeking care and date of subclade assignment were included.

## Discussion

Since the declaration of a PHEIC in August 2024 and until 23 November 2025, 82 confirmed MPXV clade I cases have been reported in 13 countries of the WHO European Region. Until October 2025, most cases were imported, with limited secondary and possibly tertiary household transmission involving partners and children. Since October 2025, an increasing number of cases without links to travel have been reported, especially among men who have sex with men, suggesting circulation of the virus in Europe [26,27]. Sexual transmission of MPXV clade Ib appears to have become an important route of transmission in Europe in recent months, especially for men who have sex with men. Experience from MPXV clade IIb suggests that the spillover risk for the general population remains limited [7].

We describe here also two cases of MPXV clade Ia infection, both with recent travel to the Democratic Republic of the Congo. One of the sequences contained a mutational signature widely reported for MPXV clade IIb virus genomes during the 2022–2023 global outbreak. The presence of this host-driven mutagenesis pattern in a MPXV clade Ia virus suggests substantial person-to-person transmission [28].

Follow-up of flight passengers was implemented inconsistently across countries, which limited assessment of transmission risk during air travel. No secondary cases were identified among reported travel contacts in available data, in line with published evidence suggesting a very low risk of MPXV transmission during air travel for MPXV clade IIb [29]. Disentangling transmission routes in household settings was complicated by the sensitive nature of the infection and the multiple possibilities of contact and fomite use. This underlines the importance of reinforcing existing WHO recommendations for household contacts [25]. The healthcare-associated case occurred before mpox was suspected clinically, when the patient was managed using precautions appropriate to an alternative working diagnosis rather than mpox-specific precautions [24]. This highlights the importance of considering mpox early in the differential diagnosis of compatible presentations in healthcare settings.

Infections with MPXV clade I have been previously considered more severe than clade II infections [9,30]. However, our data showed that reported cases in the European Region were much less severe than those reported in African countries; no deaths were observed, and among the 79 cases for whom hospitalisation status was available, only four were hospitalised for clinical reasons, including one child. This is consistent with more recent data indicating that, in areas where MPXV clade Ib has become endemic, case fatality rates have been reported to be below 1% [9]. This may reflect not only differences in the affected populations and transmission patterns, but also earlier healthcare seeking and access to supportive care. Interpretation of underlying clinical risk factors was limited by incomplete data; among cases with available information, six were reported to be living with HIV, none were reported to have weakened immune systems other than HIV, and four had received mpox vaccination before symptom onset.

Since 2023, the outbreak of MPXV clade I in the Democratic Republic of the Congo has affected younger persons, including children [10,18]. In our series, sexual contact was the most likely route of transmission for 26 of 45 imported cases. Among the 25 males who were judged to have acquired the infection sexually overseas, the sex of the most likely infecting partner was known for 17, and in 12 of these cases, the partner was a female. Of the 18 locally acquired infections of MPXV clade Ib in Europe, seven were among persons under 15 years, whereas since 2022, only 38 locally acquired cases with clade II were in this age group, despite more than 33,000 cases of clade IIb in men who have sex with men [6]. This may reflect differences between the predominantly non-sexual household transmission observed following imported MPXV clade I cases, including infections among children, and the ongoing sexual transmission of clade IIb mpox among men who have sex with men in the WHO European Region. More data are needed to see whether these differences reflect contact patterns, virologic factors, or both.

Although active follow-up of contacts supported the identification of secondary cases in Europe, healthcare-seeking and diagnostic delays, especially among index cases, may have contributed to onward transmission. Delays were substantial in our case series, with a median of 6 days from seeking care to virus subclade assignment and up to 70 days among imported cases. Clinicians in primary and acute care should consider mpox in patients with compatible symptoms regardless of travel history, and request PCR with clade-specific typing at initial testing, given autochthonous MPXV clade Ib circulation in Europe. The 4-day gap between symptom onset and seeking care among contacts of known cases was notable despite active follow-up.

The outbreak in Middle and Eastern Africa, albeit declining, is ongoing despite control efforts, including vaccination. While MPXV clade I continues to circulate in these regions, further importations into the European Region are likely. In Europe, the epidemiology of MPXV clade I is changing. Early in the observation period, most cases were imported, often following heterosexual exposure in outbreak-affected countries, with only limited onward transmission, mainly in household settings. More recently, however, an increasing number of autochthonous cases have been reported among men who have sex with men, often without clear epidemiological links to known imported cases, suggesting a shift from isolated importation events towards circulation within sexual networks such as for clade IIb. The clustering of cases linked to a sex venue in Amsterdam further supports this interpretation. Similar locally acquired MPXV clade Ib transmission among men who have sex with men and their networks was also reported in the US around the same period [31]. Uncertainties remain regarding the extent of silent importations, age-specific risk, secondary attack rates and vaccine-derived protection. Transmission of MPXV clade II has continued in Europe at lower levels of 100–300 cases a month compared with the 2022 outbreak, mainly in sexual networks among men who have sex with men [6,7]. An immediate priority in Europe remains to reduce the risk of sustained MPXV clade I transmission through early detection, interruption of transmission, and protection of groups at higher risk, in line with the WHO European Region goal of mpox elimination. As more recent MPXV clade Ib spread in Europe has been largely driven by sexual networks rather than isolated importations, understanding current immunity levels among high-risk groups is important. Published estimates suggest that mpox vaccine coverage among men who have sex with men in Europe is heterogeneous and incomplete, with ECDC citing EMIS-2024 data showing two-dose self-reported coverage ranging from 0.5% to 37.9% across European Union/European Economic Area (EU/EEA) countries, which supports continued emphasis on vaccination in higher-risk groups [32]. Other modelling analyses also point to maintenance of high-risk behaviours among men who have sex with men that should be tackled through awareness campaigns [33]. Although the PHEIC was lifted in September 2025, WHO standing recommendations [20] remain important for countries to mitigate the establishment of MPXV clade I transmission.

Continued global collaboration on genomic surveillance with subtyping, early case identification, early diagnosis and systematic contact tracing, including timely cross-border information sharing, remain essential to reduce the risk of sustained MPXV clade I transmission in Europe and to understand virological evolution [7,25]. Strengthening diagnostic capacity, including subtyping, is critical to detect and monitor emerging variants and guide appropriate public health action [28,34]; the recombinant virus reported by the United Kingdom (UK) would not have been identified without routine genomic surveillance [23]. Given the observed cases among a healthcare worker and within a network with men who have sex with men, countries in Europe should ensure robust infection prevention control in healthcare facilities and sustain engagement with high-risk communities.

Finally, renewed efforts should also ensure that vaccination, clear information on infection risk and behavioural measures to reduce it reach affected groups. These include reducing the number of partners and safer sex practices, especially for individuals who may play a disproportionately high role in transmission within sexual networks.

This analysis has several limitations. We relied on case-based information reported to WHO by countries, and data completeness and detail varied between countries. As WHO works primarily with nationally reported data, some variables were not consistently available or could not be shared in sufficient detail. Laboratory approaches, sample-taking and contact follow-up practices were not standardised across the region, and countries differed in MPXV testing algorithms, specimen types and access to clade-specific typing or sequencing. Exposure histories were based on case interviews and may therefore be affected by recall and social desirability bias, particularly for sensitive exposures. Differences in case detection, contact tracing and follow-up may also have influenced the reported distribution of transmission routes and clusters. Finally, undiagnosed mild or asymptomatic infections and unrecognised transmission chains are likely to have resulted in under-ascertainment.

## Conclusion

Most early MPXV clade I cases reported in the WHO European Region were imported and resulted in limited onward transmission, mainly in household settings. More recently, autochthonous cases among men exposed through sex with other men suggest changing transmission patterns and spread within sexual networks in Europe. Severe clinical outcomes were uncommon in this case series, with no deaths observed and few hospitalisations for clinical reasons. Continued surveillance, early diagnosis, subclade-specific testing, case investigation and contact tracing remain essential to reduce the risk of further spread and possible establishment of MPXV clade I in Europe. Targeted vaccination, risk communication and engagement with affected communities should remain central in the public health response.

## Data Availability

Data are not available due to restrictions. Interested parties can request data directly from the participating countries.

## Preprint

A preprint of the first version of the paper has been submitted to MedRxiv (MS ID: MEDRXIV/2025/342473, URL: https://www.medrxiv.org/content/10.64898/2025.12.17.25342473v1.
